# Long-term safety and disease-modifying potential of metabolic and bariatric surgery in patients with systemic lupus erythematosus

**DOI:** 10.1007/s00464-025-12557-9

**Published:** 2026-03-30

**Authors:** Xinlei Zhu, Qi Wang, Valentin Mocanu, Pattharasai Kachornvitaya, Mélissa V. Wills, Andrew Strong, Salvador Navarrete, Yung Lee, Juan S. Barajas-Gamboa, Ricard Corcelles, Matthew Kroh, Jerry Dang

**Affiliations:** 1 Digestive Disease Institute, Cleveland Clinic, Cleveland, OH USA; 2 Cleveland Clinic Lerner College of Medicine of Case Western Reserve University, Cleveland, OH USA; 3 Division of Rheumatology, Department of Medicine, MetroHealth Medical Center, Cleveland, OH USA; 4 Digestive Disease Institute, Department of General Surgery, Cleveland Clinic Abu Dhabi, Abu Dhabi, United Arab Emirates

**Keywords:** Metabolic and bariatric surgery, Systemic lupus erythematosus, Sleeve gastrectomy, Roux-en-Y gastric bypass, Disease control, TriNetX

## Abstract

**Background:**

Obesity commonly coexists with systemic lupus erythematosus (SLE), exacerbating metabolic, cardiovascular, and renal complications. Metabolic and bariatric surgery (MBS) achieves durable weight loss and improves metabolic health, but its long-term safety and potential disease-modifying effects in SLE remain unclear.

**Methods:**

A multicenter, retrospective cohort study was conducted using the TriNetX global federated research network, comprising data from over 140 healthcare organizations between 2009 and 2024. Adults with SLE who underwent sleeve gastrectomy (SG) or Roux-en-Y gastric bypass (RYGB) were compared with propensity score-matched cohorts of non-SLE patients undergoing MBS and patients with SLE who do not undergo MBS. Primary outcomes included long-term nutritional and metabolic safety. Secondary outcomes evaluated changes in immunosuppressive medication use, inflammatory markers, renal function, and mortality over a five-year follow-up.

**Results:**

Among 123,219 MBS patients, 713 (0.6%) had SLE. After matching, patients with SLE had higher risks of vitamin D deficiency (53.8% vs 45.1%, p = 0.002) and hypoalbuminemia (9.7% vs 4.7%, p = 0.001), while mortality was comparable to patients without SLE. Within the SLE cohort, RYGB was associated with higher rates of ulcer, osteoporosis, and hypoalbuminemia compared with SG. Compared with matched patients with SLE who do not undergo MBS, MBS cohort was associated with reduced use of corticosteroids (32.9% vs 47.5%), immunosuppressants (9.1% vs 22.0%), and antimalarials (11.2% vs 28.1%), lower CRP and ESR levels, improved eGFR, and lower mortality (1.4% vs 4.4%, p = 0.001).

**Conclusions:**

In this large real-world study, MBS was associated with improved disease control, renal preservation, and reduced mortality in patients with SLE, though at the expense of greater nutritional risks—particularly after RYGB. Careful patient selection and long-term multidisciplinary follow-up are essential to optimize outcomes in this complex population.

Obesity has reached epidemic proportions worldwide and is associated with substantial morbidity, including type 2 diabetes, cardiovascular disease, and impaired quality of life [1, 2]. Global rates of obesity have tripled over the past four decades, and more than 40% US adults are now affected [3, 4]. Metabolic and bariatric surgery (MBS) is the most effective intervention for achieving durable weight loss and improving obesity-related comorbidities [5, 6]. The MBS volume is now more than 280,000 annually from 158,000 cases per year in over 10 years [7, 8].

Systemic lupus erythematosus (SLE) is a chronic autoimmune disease characterized by multisystem involvement, immune dysregulation, and long-term treatment with corticosteroids and immunosuppressants [9]. Patients with SLE frequently suffer from comorbidities such as metabolic syndrome, cardiovascular disease, and renal dysfunction, many of which are exacerbated by obesity and its associated state of chronic inflammation [10, 11]. Together, the coexistence of obesity and SLE poses significant health risks [12] with obesity contributing to heightened systemic inflammation, greater disease activity, and increased need for immunosuppressive therapy in SLE. Conversely, SLE itself may predispose patients to weight gain through chronic corticosteroid use, reduced physical activity, and fatigue [12, 13].

This bidirectional relationship creates a progressive cycle that complicates disease management and may exacerbate the risk of comorbidities [14]. Yet, despite the potential of MBS to reduce weight, improve metabolic health, and reduce inflammation, evidence on its role in SLE remains scarce [15]. Current literature is limited to small case series, single-center reports, or short-term follow-up, leaving gaps in understanding the safety and long-term effects of MBS in this complex population [16, 17]. Furthermore, the relationship between MBS and disease activity in SLE patients has not been comprehensively evaluated in large-scale studies, and the comparative safety profiles of different surgical procedures remain unclear.

Given these knowledge gaps and the growing prevalence of both obesity and SLE, to address this gap, we conducted a large multicenter, propensity score-matched, retrospective cohort study using the TriNetX global research network to evaluate both the safety outcomes and potential disease-control effects of MBS in patients with SLE.

## Methods

### Data source

We used the TriNetX collaborative research database, which contains data from over 140 Global Health Care Organizations (HCOs). TriNetX is a global federated research network that provides access to real-world clinical data while ensuring patient privacy through de-identification and aggregation. The platform supports retrospective cohort studies using routine clinical care data from electronic health records. Available data include comprehensive demographics, diagnoses coded with the International Classification of Diseases, Tenth Revision, Clinical Modification (ICD-10-CM), procedures identified using ICD-10-CM or Current Procedural Terminology (CPT) codes, laboratory results classified under Logical Observation Identifiers Names and Codes (LOINC), and medications mapped to RxNorm codes.

Access to TriNetX was granted through MetroHealth Medical Center, Cleveland, OH, USA. As the analysis used only de-identified, aggregated data, the study was deemed exempt from institutional review board (IRB) review. TriNetX was selected for its large sample size, diverse patient population, and comprehensive longitudinal follow-up.

### Cohort selection and study timeline

The index date was defined as the date of MBS for surgery group, or diagnosis of SLE for non-surgery group. Patients aged 18 years or older who underwent MBS or were diagnosed with SLE between January 1, 2009, and December 31, 2024, were included. Patients with a history of organ transplantation (ICD-10-CM code: Z94) were excluded. The baseline period was defined as the 12 months prior to the index date, and follow-up extended up to 5 years post-surgery.

Patient analysis groups were analyzed as follows:

- SLE with MBS (SLE w/ MBS): Defined as patients diagnosed with SLE who subsequently underwent MBS. SLE cases were identified using ICD-10 M32, requiring at least two separate occurrences prior to surgery to ensure diagnostic accuracy. MBS procedures were identified using CPT 43775 (laparoscopic sleeve gastrectomy, SG) and 43,644 (laparoscopic Roux-en-Y gastric bypass with gastroenterostomy, RYGB, Roux limb ≤ 150 cm). Only primary MBS procedures were evaluated. Inclusion criteria required the MBS to occur at least three months following the SLE diagnosis to ensure established disease management and exclude patients with more recent diagnoses.
- SLE without MBS (SLE w/o MBS): Defined as patients with SLE who did not undergo MBS. Inclusion required at least two occurrences of SLE diagnosis (ICD-10 M32) to ensure diagnostic accuracy and exclude single diagnostic codes that may represent rule-out diagnoses. Patients with any documented MBS (CPT 43775, 43,644, or ICD-10 Z98.84) were excluded.
- MBS without SLE (MBS w/o SLE): Comprised patients without SLE who underwent primary MBS. This cohort was identified through the presence of CPT codes 43,644 or 43,775. Any patient with a diagnosis of SLE (ICD-10-CM M32) was excluded.

### Outcome variables collected

#### Primary outcomes—long-term nutritional and metabolic outcomes

Long-term safety outcomes were assessed from post-surgery day 1 to 5 years and identified using ICD-10 codes for gastrointestinal hemorrhage (K92.2), vitamin D deficiency (E55), vitamin B12 or B9 deficiency (E53.8), iron deficiency (E61.1), calcium deficiency (E58), albumin/protein deficiency (E46, E88.09), osteoporosis (M81), gastrointestinal ulcers (K28), osteopenia (M89), body mass index (BMI), and mortality.

#### Secondary outcomes—SLE disease-modifying outcomes

These included changes in the newly prescribed use of immunosuppressive agents, hydroxychloroquine, and markers of disease activity.

Medications assessed included:

- Glucocorticoids: prednisone (8640), methylprednisolone (6902), hydrocortisone (5492)
- Conventional immunosuppressants: mycophenolate mofetil (6183), azathioprine (1256), methotrexate (6851), cyclophosphamide (3008), tacrolimus (11,170), leflunomide (27,169)
- Antimalarial: hydroxychloroquine (5521)
- Biologics: belimumab (1,155,962), rituximab (121,191), anifrolumab (2,572,829)

Additional disease-control indicators included chronic kidney disease (N18), inflammatory markers such as C-reactive protein (CRP) and erythrocyte sedimentation rate (ESR), and estimated glomerular filtration rate (eGFR). Mortality was also evaluated.

### Statistical approach to matching and cohort comparisons

Baseline characteristics were summarized using descriptive statistics. Categorical variables were reported as percentages, while continuous variables were expressed as mean ± standard deviation (SD). Comparisons between groups were performed using t-tests for continuous variables and chi-squared analysis for categorical variables.

To reduce potential confounding, 1:1 propensity score matching was performed using a greedy nearest-neighbor algorithm without replacement with a caliper width of 0.1 standard deviations of the logit of the propensity score. For comparisons between patients with SLE who underwent MBS and non-SLE surgical patients, matching was based on core demographic variables including age, sex, race, BMI, and key comorbidities (type 2 diabetes, hypertension, obstructive sleep apnea (OSA), chronic obstructive pulmonary disease (COPD), hyperlipidemia, chronic kidney disease (CKD), myocardial infarction, tobacco use, and MBS type). During propensity score matching, patients for whom no suitable comparator with sufficiently similar baseline characteristics could be identified within the predefined matching framework were excluded, resulting in a reduced matched cohort.

For analyses comparing SLE patients who underwent MBS with those who did not, matching additionally included immunosuppressive medication use (glucocorticoids, mycophenolate, azathioprine, methotrexate, cyclophosphamide, tacrolimus, leflunomide, hydroxychloroquine, chloroquine, belimumab, rituximab, anifrolumab) as proxies for disease severity. Standardized differences before and after matching are reported for all covariates.

Statistical significance was set an adjusted p < 0.05. All analyses were performed using the built-in statistical tools within the TriNetX platform. Due to limitations of the TriNetX database, subgroup analyses could only be performed among patients who underwent either SG or RYGB exclusively within the study period.

## Result

### Cohort summary for long-term safety outcome

For safety outcomes, a total of 123,219 patients underwent MBS during the study period, including 713 (0.6%) patients with SLE and 122,506 (99.4%) patients without SLE. After propensity score matching, 621 patients remained in each group for comparative analysis. Median follow-up time was 1,244 days (IQR: 1,172 days) for the SLE cohort and 1,284 days (IQR: 1,116 days) for the non-SLE cohort.

### Baseline characteristics of SLE w/ MBS vs. MBS w/o SLE cohorts

Before matching, patients with SLE were older at the time of surgery and predominantly female. They had higher prevalence of comorbidities including tobacco use, type 2 diabetes mellitus, OSA, hypertension, COPD, hyperlipidemia, CKD, and prior myocardial infarction. Patients with SLE also demonstrated substantially higher use of immunosuppressive medications, antimalarials, and corticosteroids compared to patients without SLE. Although statistically significant, BMI differences between the two groups may not be clinically significant (Table 1).

**Table 1:** Basic characteristics compare between SLE w/ MBS and MBS w/o SLE

Variable	SLE w/ MBS n = 713	MBS w/o SLE n = 122,506	p-value	SLE subgroup analysis		
				SLE SG n = 464	SLE RYGB n = 223	p-value
BMI (kg/m^2^)	44.1 ± 7.4	45.0 ± 7.6	0.005	44.1 ± 7.7	44.3 ± 7.8	0.728
Age at index (years)	50.4 ± 10.7	43.4 ± 12.1	< 0.001	52.1 ± 9.9	49.8 ± 10.9	0.009
White	357 (50.1%)	76,860 (62.7%)	< 0.001	224 (48.3%)	119 (53.4%)	0.211
Female	671 (94.1%)	95,144 (77.7%)	< 0.001	439 (94.6%)	208 (93.3%)	0.483
Black or African American	234 (32.8%)	25,563 (20.9%)	< 0.001	164 (35.3%)	62 (27.8%)	0.047
Tobacco use	41 (5.8%)	4,622 (3.8%)	0.006	13 (2.8%)	10 (4.5%)	0.251
Type 2 diabetes mellitus	250 (35.1%)	37,922 (31.0%)	0.018	141 (30.4%)	72 (32.3%)	0.614
Sleep apnea	475 (66.6%)	68,892 (56.2%)	< 0.001	294 (63.4%)	138 (61.9%)	0.707
Hypertension	533 (74.8%)	69,886 (57.0%)	< 0.001	320 (69.0%)	154 (69.1%)	0.95
COPD	75 (10.5%)	4,889 (4.0%)	< 0.001	35 (7.5%)	25 (11.2%)	0.302
Hyperlipidemia	313 (43.9%)	44,678 (36.5%)	< 0.001	171 (36.9%)	87 (39.0%)	0.584
Acute myocardial infarction	30 (4.2%)	1,517 (1.2%)	< 0.001	10* (2.2%)	10* (4.5%)	0.089
CKD	103 (14.4%)	5,087 (4.2%)	< 0.001	65 (14.0%)	21 (9.4%)	0.089
Gastric bypass	234 (32.8%)	45,455 (37.1%)	0.018	0 (0.0%)	223 (100.0%)	< 0.001
Sleeve gastrectomy	490 (68.7%)	77,475 (63.2%)	0.002	464 (100.0%)	0 (0.0%)	< 0.001
**Medication**						
Methotrexate	155 (21.7%)	1,307 (1.1%)	< 0.001	41 (8.8%)	18 (8.1%)	0.738
Hydroxychloroquine	471 (66.1%)	1,839 (1.5%)	< 0.001	206 (44.4%)	93 (41.7%)	0.565
Leflunomide	40 (5.6%)	264 (0.2%)	< 0.001	13 (2.8%)	10* (4.5%)	0.251
Rituximab	23 (3.2%)	159 (0.1%)	< 0.001	10* (2.2%)	0 (0%)	0.027
Prednisone	442 (62.0%)	22,152 (18.1%)	< 0.001	129 (27.8%)	63 (28.3%)	0.902
Azathioprine	107 (15.0%)	229 (0.2%)	< 0.001	32 (6.9%)	15 (6.7%)	0.934
Cyclophosphamide	10* (1.4%)	190 (0.2%)	< 0.001	0 (0.0%)	0 (0.0%)	0.000
Tacrolimus	24 (3.4%)	672 (0.5%)	< 0.001	10* (2.2%)	10* (4.5%)	0.089
Anifrolumab	10* (1.4%)	0 (0.0%)	< 0.001	0 (0.0%)	10* (4.5%)	< 0.001
Belimumab	66 (9.3%)	10* (0.0%)	< 0.001	29 (6.2%)	14 (6.3%)	0.989
Methylprednisolone	378 (53.0%)	25,405 (20.7%)	< 0.001	108 (23.3%)	60 (26.9%)	0.300
Hydrocortisone	198 (27.8%)	11,607 (9.5%)	< 0.001	51 (11.0%)	24 (10.8%)	0.928

*SLE* Systemic lupus erythematosus,* MBS* Metabolic and bariatric surgery,* SG* Sleeve gastrectomy,* RYGB* Roux-en-Y gastric bypass,* BMI* Body mass index,* SD* Standard deviation,* COPD* Chronic obstructive pulmonary disease,* eGFR* Estimated glomerular filtration rate. To protect patient privacy, numbers < 10 are rounded up to 10. Subgroup totals do not sum to the full RA w/ MBS cohort due to analysis restrictions within TriNetX (see Methods)

### Long-term safety outcomes

In the matched comparison for long-term safety outcome, patients with SLE had higher rates of vitamin D deficiency (53.8% vs 45.1%, p = 0.002) and hypoalbuminemia (9.7% vs 4.7%, p = 0.001) compared with patients without SLE. Vitamin B12 or B9 deficiency (21.3% vs 17.2%, p = 0.072), iron deficiency (13.2% vs 10.6%, p = 0.161), and gastrointestinal ulcer (4.2% vs 2.4%, p = 0.081) did not reach statistical significance after multiple comparison correction. Rates of osteoporosis (6.8% vs 5.0%, p = 0.184), osteopenia (both 5.8%, p = 1.000), gastrointestinal hemorrhage (1.8% vs 1.6%, p = 0.826), and mortality (both 1.6%, p = 1.000) were comparable between groups (Table 2).

**Table 2:** Safety outcome compare between SLE w/ MBS and MBS w/o SLE

Outcome	SLE w/ MBS n = 621	MBS w/o SLE n = 621	RR	95% CI	p-value	SLE subgroup analysis		
						SLE SG n = 464	SLE RYGB n = 223	p-value
BMI (kg/m^2^)	34.6 ± 7.7	35.0 ± 7.8			0.398	35.3 ± 7.8	32.9 ± 7.5	0.001
Vitamin B12 and/ or B9 deficiency	132 (21.3%)	107 (17.2%)	1.234	0.981‚ 1.552	0.072	93 (20.0%)	57 (25.6%)	0.101
Vitamin D deficiency	334 (53.8%)	280 (45.1%)	1.193	1.065‚ 1.336	0.002	254 (54.7%)	121 (54.3%)	0.906
Gastrointestinal hemorrhage	10* (1.6%)	10* (1.6%)	1.000	0.419‚ 2.386	1.000	10* (2.2%)	10* (4.5%)	0.089
Iron deficiency	82 (13.2%)	66 (10.6%)	1.242	0.916‚ 1.685	0.161	55 (11.9%)	33 (14.8%)	0.280
Albumin/protein deficiency	60 (9.7%)	29 (4.7%)	2.069	1.347‚ 3.178	0.001	39 (8.4%)	30 (13.5%)	0.039
Osteoporosis	42 (6.8%)	31 (5.0%)	1.355	0.863‚ 2.126	0.184	25 (5.4%)	24 (10.8%)	0.010
Gastrojejunal ulcer	26 (4.2%)	15 (2.4%)	1.733	0.927‚ 3.241	0.081	10* (2.2%)	27 (12.1%)	0.001
Osteopenia	36 (5.8%)	36 (5.8%)	1.000	0.639‚ 1.566	1.000	26 (5.6%)	14 (6.3%)	0.724
Death	10* (1.6%)	10* (1.6%)	1.000	0.419‚ 2.386	1.000	10* (2.2%)	10* (4.5%)	0.089

*SLE* Systemic lupus erythematosus;* MBS* Metabolic and bariatric surgery,* SG* Sleeve gastrectomy,* RYGB* Roux-en-Y gastric bypass,* BMI* Body mass index,* SD* Standard deviation,* COPD* Chronic obstructive pulmonary disease,* RR* Risk ratio,* CI* Confidence interval. To protect patient privacy, numbers < 10 are rounded up to 10. Subgroup totals do not sum to the full RA w/ MBS cohort due to analysis restrictions within TriNetX (see Methods)

### Subgroup analysis: SLE SG vs. SLE RYGB

Within the SLE cohort (SG n = 464; RYGB n = 223), RYGB was associated with higher rates of gastrointestinal ulcer (12.1% vs 2.2%, p < 0.001), osteoporosis (10.8% vs 5.4%, p = 0.010), and hypoalbuminemia (13.5% vs 8.4%, p = 0.039). Vitamin D deficiency was similar between procedures (54.7% vs 54.3%, p = 0.906). Vitamin B12 or B9 deficiency (25.6% vs 20.0%, p = 0.101) and gastrointestinal hemorrhage (4.5% vs 2.2%, p = 0.089) were numerically higher after RYGB, but differences were not statistically significant. Osteopenia (6.3% vs 5.6%, p = 0.724) and mortality (4.5% vs 2.2%, p = 0.089) were comparable between procedures (Table 2).

### Cohort summary for SLE disease-modifying outcome

For disease-control outcomes, a total of 205,734 patients were identified during the selected period, including 713 (0.3%) patients with SLE who underwent MBS and 205,021 (99.7%) patients with SLE who did not undergo MBS. After propensity score matching, 621 patients remained in each group for comparative analysis. Median follow-up time was 1,187 days (IQR: 1,222 days) for the MBS cohort and 1,444 days (IQR: 1,140 days) for the non-MBS cohort.

### Baseline characteristics of SLE w/ MBS vs SLE w/o MBS cohorts

Before matching, patients with SLE who underwent MBS were predominantly female. They had higher prevalence of comorbidities including tobacco use, type 2 diabetes mellitus, sleep apnea, hypertension, COPD, hyperlipidemia, chronic kidney disease, and prior myocardial infarction. Patients with SLE who underwent MBS also demonstrated substantially higher use of immunosuppressive medications, antimalarials, and corticosteroids compared to patients without MBS, and BMI is significantly higher in the MBS group. Full details are provided in Table 3.

**Table 3:** Basic characteristics compare between SLE w/ MBS and SLE w/o MBS

Variable	SLE w/ MBS n = 713	SLE w/o MBS n = 205,021	p-value	SLE subgroup analysis		
				SLE SG n = 464	SLE RYGB n = 223	p-value
BMI (kg/m^2^)	44.1 ± 7.3	29.0 ± 7.8	< 0.001	44.1 ± 7.7	44.3 ± 7.8	0.728
White	357 (50.1%)	100,542 (49.0%)	0.583	224 (48.3%)	119 (53.4%)	0.211
Female	671 (94.1%)	178,704 (87.2%)	< 0.001	439 (94.6%)	208 (93.3%)	0.483
Black or African American	234 (32.8%)	44,771 (21.8%)	< 0.001	164 (35.3%)	62 (27.8%)	0.047
Tobacco use	19 (2.7%)	2532 (1.2%)	0.001	13 (2.8%)	10* (4.5%)	0.251
Type 2 diabetes mellitus	219 (30.7%)	18,289 (8.9%)	< 0.001	141 (30.4%)	72 (32.3%)	0.614
Sleep apnea	443 (62.1%)	9071 (4.4%)	< 0.001	294 (63.4%)	138 (61.9%)	0.707
Hypertension	493 (69.1%)	53,221 (26.0%)	< 0.001	320 (69.0%)	154 (69.1%)	0.950
COPD	59 (8.3%)	8432 (4.1%)	< 0.001	35 (7.5%)	25 (11.2%)	0.302
Hyperlipidemia	263 (36.9%)	21,498 (10.5%)	< 0.001	171 (36.9%)	87 (39.0%)	0.584
Acute myocardial infarction	13 (1.8%)	2410 (1.2%)	0.109	10* (2.2%)	10* (4.5%)	0.089
CKD	87 (12.2%)	15,539 (7.6%)	< 0.001	65 (14.0%)	21 (9.4%)	0.089
**Medication**						
Methotrexate	60 (8.4%)	6788 (3.3%)	< 0.001	41 (8.8%)	18 (8.1%)	0.738
Hydroxychloroquine	310 (43.5%)	50,022 (24.4%)	< 0.001	206 (44.4%)	93 (41.7%)	0.565
Leflunomide	20 (2.8%)	1400 (0.7%)	< 0.001	13 (2.8%)	10 (4.5%)	0.251
Rituximab	10* (1.4%)	823 (0.4%)	< 0.001	10* (2.2%)	0 (0%)	0.027
Prednisone	200 (28.1%)	35,766 (17.4%)	< 0.001	129 (27.8%)	63 (28.3%)	0.902
Azathioprine	50 (7.0%)	5525 (2.7%)	< 0.001	32 (6.9%)	15 (6.7%)	0.934
Cyclophosphamide	0 (0.0%)	870 (0.4%)	0.081	0 (0.0%)	0 (0.0%)	0.000
Tacrolimus	10* (1.4%)	1233 (0.6%)	0.006	10* (2.2%)	10* (4.5%)	0.089
Anifrolumab	10* (1.4%)	51 (0.0%)	< 0.001	0 (0.0%)	10* (4.5%)	< 0.001
Belimumab	45 (6.3%)	1310 (0.6%)	< 0.001	29 (6.2%)	14 (6.3%)	0.989
Methylprednisolone	176 (24.7%)	20,166 (9.8%)	< 0.001	108 (23.3%)	60 (26.9%)	0.300
Hydrocortisone	79 (11.1%)	6788 (3.3%)	< 0.001	51 (11.0%)	24 (10.8%)	0.928
eGFR	84.5 ± 25.7	82.7 ± 33.3	0.162	85.1 ± 26.5	82.6 ± 24.1	0.245

*SLE* Systemic lupus erythematosus,* MBS* Metabolic and bariatric surgery,* SG* Sleeve gastrectomy,* RYGB* Roux-en-Y gastric bypass,* BMI* Body mass index,* SD* Standard deviation,* COPD* Chronic obstructive pulmonary disease,* eGFR* Estimated glomerular filtration rate. To protect patient privacy, numbers < 10 are rounded up to 10. Subgroup totals do not sum to the full RA w/ MBS cohort due to analysis restrictions within TriNetX (see Methods)

### Disease-modifying outcomes

In the matched analysis, patients with SLE who underwent MBS had lower rates of conventional immunosuppressant use (9.1% vs 22.0%, p < 0.001), antimalarial use (11.2% vs 28.1%, p < 0.001), and corticosteroid use (32.9% vs 47.5%, p = 0.003) compared with matched patients with SLE without MBS. CKD (5.3% vs 13.8%, p < 0.001) and osteoporosis (4.1% vs 7.2%, p = 0.013) were also less frequent in the MBS group. Mortality was lower among patients with SLE who underwent MBS (1.4% vs 4.4%, p = 0.001). Laboratory measures showed lower CRP (5.8 ± 6.7 vs 8.2 ± 8.9 mg/L, p = 0.002), lower ESR (18.3 ± 13.4 vs 22.7 ± 16.2 mm/hr, p = 0.001), and higher eGFR (92.4 ± 15.8 vs 87.1 ± 17.3 mL/min/1.73 m2, p < 0.001) in the MBS group (Table 4).

**Table 4:** SLE disease-control outcomes

outcome	patient at risk	SLE w/ MBS	patient at risk	SLE w/o MBS	RR	95% CI	P value
Conventional immunosuppressants	419	38 (9.1%)	527	116 (22.0%)	0.412	0.292‚ 0.581	< 0.001
Biologic agents	613	32 (5.2%)	655	49 (7.5%)	0.698	0.453‚ 1.074	0.100
Corticosteroid	164	54 (32.9%)	280	133 (47.5%)	0.693	0.539‚ 0.891	0.003
Osteoporosis	661	27 (4.1%)	664	48 (7.2%)	0.565	0.357‚ 0.894	0.013
CKD	601	32 (5.3%)	602	83 (13.8%)	0.386	0.261‚ 0.571	< 0.001
Antimalarials	241	27 (11.2%)	385	108 (28.1%)	0.399	0.270‚ 0.590	< 0.001
Death	703	10* (1.4%)	703	31 (4.4%)	0.323	0.159‚ 0.653	0.001
CRP (mg/L)	703	5.8 ± 6.7	703	8.2 ± 8.9			0.002
ESR (mm/hr)	703	18.3 ± 13.4	703	22.7 ± 16.2			0.001
eGFR (mL/min/1.73 m^2^)	703	92.4 ± 15.8	703	87.1 ± 17.3			< 0.001

*SLE* Systemic lupus erythematosus, *MBS* Metabolic and bariatric surgery, *eGFR* estimated glomerular filtration rate; *CI* Confidence interval. Conventional immunosuppressants, mycophenolate mofetil, azathioprine, methotrexate, cyclophosphamide, tacrolimus, leflunomide; Biologic agents: belimumab, rituximab, anifrolumab; Cocorticoids: prednisone, methylprednisolone, hydrocotisol; Antimalarial: hydroxychloroquine. To protect patient privacy, numbers < 10 are rounded up to 10

### Subgroup analysis SLE SG vs SLE RYGB

Within the SLE cohort, disease-modifying outcomes were generally comparable between procedures. Rates of conventional immunosuppressant use were 8.5% in SG and 8.1% in RYGB (p = 0.883). Corticosteroid use was lower in SG (28.8%) than in RYGB (41.7%), but this did not reach statistical significance (p = 0.113). CKD was reported in 3.8% of SG vs. 7.2% of RYGB (p = 0.080), and osteoporosis in 3.6% vs 5.9% (p = 0.180). CRP, ESR, and eGFR values were similar between procedures (Table 5).

**Table 5:** SLE disease-control subgroup analysis

Outcome	Patient at risk	SLE w/ MBS	Patient at risk	SLE w/o MBS	RR	95% CI	p-value
Conventional immunosuppressants	270	23 (8.5%)	136	11 (8.1%)	1.053	0.529‚ 2.096	0.883
Biologic agents	402	21 (5.2%)	190	10* (5.3%)	0.993	0.477‚ 2.066	0.984
Corticosteroid	111	32 (28.8%)	48	20 (41.7%)	0.692	0.444‚ 1.079	0.113
Osteoporosis	442	16 (3.6%)	202	12 (5.9%)	0.609	0.294‚ 1.264	0.18
CKD	390	15 (3.8%)	195	14 (7.2%)	0.536	0.264‚ 1.087	0.08
Antimalarials	152	16 (10.5%)	81	10* (12.3%)	0.853	0.406‚ 1.792	0.674
Death	464	10* (2.2%)	223	10* (4.5%)	0.481	0.203‚ 1.138	0.089
CRP (mg/L)	464	10.46 ± 19.64	223	15.16 ± 36.29			0.165
ESR (mm/hr)	464	25.64 ± 21.69	223	23.24 ± 19.45			0.374
eGFR (mL/min/1.73 m^2^)	464	86.11 ± 26.83	223	87.04 ± 25.88			0.678

*SLE* Systemic lupus erythematosus,* MBS* Metabolic and bariatric surgery, * eGFR* estimated glomerular filtration rate, * CI* Confidence interval. Conventional immunosuppressants, mycophenolate mofetil, azathioprine, methotrexate, cyclophosphamide, tacrolimus, leflunomide; Biologic agents: belimumab, rituximab, anifrolumab; Corticoids: prednisone, methylprednisolone, hydrocortisone; Antimalarial: hydroxychloroquine. To protect patient privacy, numbers < 10 are rounded up to 10

## Discussion

In this large, multicenter, real-world cohort study using the TriNetX network, we evaluated long-term safety and disease-control outcomes of MBS in patients with SLE. To our knowledge, this is the first study to investigate these outcomes in a worldwide, propensity score-matched analysis. We found that MBS was associated with improved disease control, reduced of medication requirements, and lower rates of comorbidities, and also with increased nutritional risks, particularly after RYGB.

A major strength of this study is the use of a large, federated, real-world database capturing over 140 HCOs, which provides diverse and generalizable findings. The application of propensity score matching allowed for balanced comparisons between SLE and non-SLE populations, as well as between different surgical approaches, minimizing confounding from baseline demographic and comorbidity differences. The longitudinal design with up to five years of follow-up offers novel insights into long-term outcomes, which are rarely reported in SLE populations undergoing MBS. Additionally, the application of multiple comparison corrections strengthens the reliability of our statistical inferences.

Our findings suggest that MBS may be associated with effective improvements with disease-control indicators in patients with SLE. Compared with matched controls, patients undergoing MBS demonstrated significantly lower use of conventional immunosuppressants, corticosteroids, and antimalarials. In addition, reductions in inflammatory markers such as CRP and ESR were observed, alongside preservation of kidney function as indicated by higher eGFR levels. These changes may be clinically meaningful, as they likely reflect decreased disease activity, reduced treatment burden, and potentially ultimately improved quality of life in this population. However, it is important to note that medication usage patterns may not fully reflect clinical disease activity, as prescribing practices can be influenced by factors beyond disease severity, including prophylactic use and physician preferences.

The mechanisms underlying the observed associations between MBS and improved disease-control indicators in SLE are multifactorial and likely revolve around the complex immuno-metabolic changes associated with metabolic surgery. Weight loss itself reduces many adipose tissue-derived pro-inflammatory cytokines such as TNF-α and Interleukin-6 (IL-6), thereby potentially lowering systemic inflammation [18–20]. Improved insulin sensitivity and metabolic homeostasis after surgery may further modulate immune responses and reduce autoimmunity [21, 22]. Additionally, MBS may improve gastrointestinal health through modifications in gut microbiota and metabolic byproducts including short-chain fatty acids and bile salts, potentially contributing to reduced systemic inflammation through the gut-immune axis. A narrative review of rheumatic diseases suggests that surgical weight loss may favorably modify disease trajectories across conditions such as rheumatoid arthritis and gout, although concerns about bone health like osteopenia and osteoporosis persist [23]. However, these proposed mechanisms remain largely hypothetical in the context of SLE, and our observational study design cannot establish causality.

Despite these encouraging disease-control results, safety outcomes underscore the importance of careful perioperative and postoperative management. Patients with SLE were more likely to experience vitamin D deficiency and protein-calorie malnutrition. Subgroup analysis demonstrated that RYGB carried higher risks of gastrojejunal ulcer, osteoporosis, and hypoalbuminemia as compared to SG. These findings support SG as an option for SLE patients, particularly for those already at risk of bone disease or nutritional compromise such as patients on, or expected to require long-term disease-modifying antirheumatic drugs. The higher ulcer risk with RYGB may be particularly concerning in SLE patients who often require anti-inflammatory medications that can further increase gastrointestinal complications.

Immune dysregulation and chronic immunosuppressive therapy in SLE predispose patients to postsurgical complications, while MBS may exacerbate the malabsorptive complications like vitamin D deficiency [24]. Vitamin D deficiency is also particularly relevant in SLE, as low 25(OH)D levels are common in this population due to photosensitivity, sun avoidance, and medication effects. Prior studies have shown that up to 70–90% of patients with SLE are vitamin D deficient, and lower serum vitamin D levels are significantly associated with higher disease activity scores and organ damage indices [25, 26]. Corcelles et al. reported a 12.9% early major postoperative complication rate, significantly associated with immunosuppressant use [16]. A retrospective analysis of the U.S. Nationwide Inpatient Sample further demonstrated that among hospitalized patients with SLE, a history of MBS was associated to lower risks of SLE-related complications but a higher likelihood of malnutrition [15]. Similarly, Hefler and colleagues, using the MBSAQIP database, found that immunocompromised patients faced greater risks of major complications after bariatric surgery, particularly with RYGB [17]. However, these data were limited to short-term outcomes (within 30 days postoperatively), whereas our study provides longer follow-up and more comprehensive medication-related information.

This study has several important limitations. First, disease control was assessed using medication patterns and selected laboratory markers, which may not fully capture clinical disease activity or reflect standardized disease activity indices such as SLEDAI or BILAG. The use of medication patterns as surrogate markers for disease control is problematic as it may be influenced by confounding by indication, where sicker patients receive more intensive treatment regardless of surgical intervention. Second, outcomes were identified through ICD-10 and CPT codes, which are subject to incomplete capture and potential misclassification. Although validation studies suggest good positive predictive value for SLE diagnosis codes, misclassification bias cannot be entirely excluded. Third, follow-up was limited to within five years, and longer observation is needed to evaluate durability of benefits and late complications. Fourth, the observational design precludes causal inferences, and residual confounding from unmeasured variables such as disease severity, socioeconomic factors, and healthcare access may persist despite propensity score matching. Fifth, selection bias may have influenced which SLE patients were deemed suitable surgical candidates, potentially leading to outcomes that may not be generalizable to all SLE patients with obesity. Sixth, MBS is delivered within a multidisciplinary care framework, and the independent effects of dietary, medical, and psychological interventions could not be evaluated. In addition, procedure-specific outcomes such as revision or reoperation were not reliably captured due to database limitations. Finally, missing data on important clinical variables such as specific disease activity scores and detailed medication dosages limit the depth of our analysis.

Despite these limitations, our findings provide new and important insights for clinicians caring for obese patients with SLE. Key questions we have answered include whether MBS surgery is safe in SLE patients—our data suggest it is, with comparable mortality to non-SLE controls. While we cannot definitively answer which type of MBS is optimal, patients expected to require high doses of disease-modifying antirheumatic drugs or chronic corticosteroids may benefit from SG to avoid marginal ulcers, while those who do undergo RYGB may benefit from more intensive nutritional monitoring and supplementation.

## Conclusion

In this large-scale real-world analysis of over 123,000 patients, MBS was associated with meaningful improvements in disease-control indicators for patients with SLE, including reduced immunosuppressive medication requirements, lower inflammatory markers, preserved renal function, and lower mortality rates, but at the cost of increased nutritional complications, particularly vitamin D deficiency and hypoalbuminemia. Both SG and RYGB for patients with SLE demonstrated acceptable mortality rates comparable to non-SLE controls, suggesting that carefully selected obese patients with SLE can safely undergo MBS with appropriate perioperative management and long-term multidisciplinary monitoring.
